# Rotational atherectomy via the transradial access: success rates, procedural parameters and complications

**DOI:** 10.1007/s00380-022-02053-8

**Published:** 2022-03-19

**Authors:** Paul Ferstl, Anne-Sophie Drentwett, Sophie Bargon, Nora Schacher, Monique Tröbs, Mohamed Marwan, Stephan Achenbach, Luise Gaede

**Affiliations:** Department of Cardiology, Friedrich-Alexander-Universität (FAU) Erlangen-Nürnberg, Ulmenweg 18, 91054 Erlangen, Germany

**Keywords:** Rotational atherectomy, Transradial, Percutaneous coronary intervention, Radial access

## Abstract

Radial access is recommended for percutaneous coronary intervention (PCI), but rotational atherectomy remains frequently performed via femoral access. Analyzing the procedural parameters, success rate and complications of rotational atherectomy, performed via radial in comparison to femoral access. We retrospectively analyzed 427 consecutive patients undergoing rotational atherectomy. Procedural parameters and outcome were determined in 171 patients, scheduled for radial and compared to 256 patients with femoral access use. In the radial access group (74 ± 9 years, 84% male), the LAD was most frequently treated (49%). Sheath size was 7F in 59% and 6F in 41%, burr size was 1.5 mm in 46% and 1.25 mm in 14% of patients. A temporary pacemaker was inserted in 14%. Procedural success rate stood at 97%. Access site complications occurred in 4% of patients, which was significantly less frequent than in in 256 patients treated via femoral access (13% *p* = 0.003). Compared to radial access, femoral access was associated with the use of larger sheaths (*p* < 0.001), more frequent treatment of non-LAD vessels (58.2% vs. 44.4%, *p* = 0.013) and a higher rate of temporary pacemaker use (27%; *p* = 0.001). No differences could be seen in procedural success (*p* = 0.83) and burr size (*p* = 0.51). Femoral access (OR 3.33; 95% CI 1.40–7.93), and female sex (OR3.40 95% CI 1.69–6.63) were independent predictors for access site complications. For coronary rotational atherectomy, radial access has a high success rate with overall use of smaller sheaths, but of equally sized burrs as well as a significant lower rate of access site-related complications than femoral access.

## Introduction

To guarantee sufficient stent expansion in patients with heavily calcified coronary stenoses, calcium modification is crucial. Rotational atherectomy represents one of the established tools to modify calcified lesions [1, 2]. While the radial approach is recommended as the gold standard for percutaneous coronary intervention (PCI), recommendations concerning its use in case of rotational atherectomy borders on the cautious side in both European as well as North American expert consensus documents [1, 2]. Concerns regarding sheath and guiding catheter size, but also successful delivery of the burr, as well as the need for placement of a temporary pacemaker play an important role in the former, and most likely explain why the use femoral access remains dominant [3, 4].

In the largest registry to date, which compares radial and femoral access in over 8000 patients undergoing rotational atherectomy, patients treated via radial access showed equal 30-day mortality, but lower rates of major bleeding and access site complications [4], whereas data from a big propensity-matched cohort do not confirm these findings [5]. Research that reports detailed procedural data in patients undergoing rotational atherectomy via transradial versus transfemoral is minimal and builds on uniformly small sample groups [6–8]. However, such data are necessary before a general recommendation to perform rotational atherectomy via the radial access can be made.

We, thus, systematically analyzed procedural data in patients undergoing rotational atherectomy via radial access, and compared baseline parameters and procedural data with patients undergoing rotational atherectomy via femoral access in a high-volume center.

## Methods

### Patients

We retrospectively included all patients in whom PCI with rotational atherectomy was intended either ad hoc, following coronary angiography, or as a scheduled elective procedure between 03/2013 and 06/2021. The procedure was performed using either the RotaLink™ (until Dec 2018, *n* = 192) or the RotaPro™ rotational atherectomy system (*n* = 235), both from Boston Scientific Corporation, Marlborough, Massachusetts, USA. All operators were certified by the manufacturer. Either the Rota Floppy, or the Rota ExtraSupport (ES) wire was used for the rotational atherectomy. Choices regarding access site, wire type and burr size were at the operators’ discretion.

Patients were divided depending on the access site for performing rotational atherectomy into a radial or a femoral group. Accordingly, patients who received rotational atherectomy followed by PCI via radial access, but also had femoral venous access for a temporary pacer were assigned to the radial group. To accommodate for changes in the preferred access route over the time of the observation interval, three equally large patient cohorts were separated in three groups of almost equal size: Patients treated in 2013–2017, in 2018–2019 and in 2020–08/2021.

After a primary analysis of baseline criteria and procedural characteristics including sheath and guiding sizes, patients in whom rotational atherectomy had been intended but was not finally performed, e.g., due to inability to cross the lesion with a Rota wire or to advance the burr to the stenosis, were excluded for further periprocedural analysis. This analysis included sheath and guiding size as well, and added burr size, stent length, use of temporary pacemaker and periprocedural complications. In patients with the use of two or more sheaths, guiding catheters or burrs, the largest size was used for the analysis.

### Definitions

Procedural success of rotational atherectomy was defined as the possibility to perform rotational atherectomy, to treat the vessel successfully either with a stent implantation or with the application of a drug-eluting balloon, resulting in a residual stenosis of less than 10% and TIMI III flow, without any relevant dissection in the treated vessel, determined by the final cine angiogram.

Periprocedural complications were defined as coronary artery perforation (including perforation of Ellis types I–III as well as cavity-spilling perforations), temporary or permanent slow- or no-reflow phenomena, hemodynamic instability needing medication, relevant bradycardia needing medication or emergency insertion of a temporary pacemaker, relevant ischemic ECG changes defined as new ST-segment elevation or depression accompanied by angina, device complications (e.g., the inability to retract the burr or wire break) and periprocedural death.

Access-related complications were evaluated for all patients scheduled for rotational atherectomy except in those patients in whom percutaneous circulatory support was additionally placed via the transfemoral access. Access-related complications were defined as a composite of bleeding, aneurysm, AV fistula and peripheral ischemia. Bleeding was defined using the Bleeding Academic Research Consortium (BARC). [9] Additionally, any potential drop of hemoglobin (HB) as well as the need for transfusions of any kind were evaluated for the patients with a bleeding complication.

### Statistical analysis

Presented data are shown in either mean and standard deviation (mean ± SD) or when continuous data are concerned, in median with interquartile ranges (IQR), while nominal variables are shown as percentages. Categorical variables were compared using Pearson’s chi-square test while continuous variables were tested either using the t-test or using the Mann–Whitney U test depending on the distribution of the parameters. A *p*-value ≤ 0.05 was considered statistically significant. Binary logistic regression analysis was used to identify predictors of access-related complications and to determine odds ratios (OR) in univariate analysis. For all statistical analyses, we used IBM SPSS Statistics Version 24.

## Results

### Baseline characteristics

**Fig. 1:**
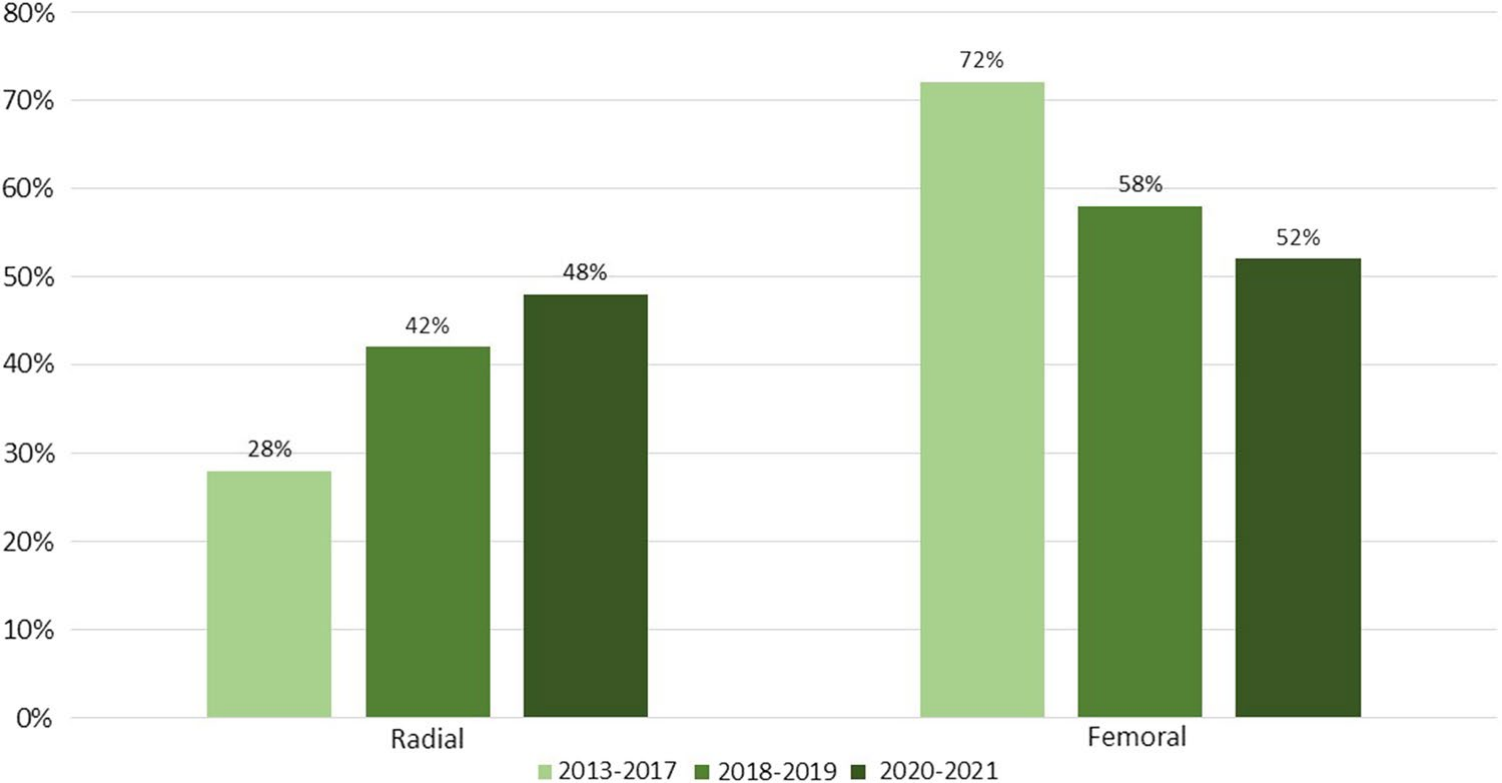
Change in access site within the years 2013–2017, 2018–2019, 2020–2021

In total, we included 427 patients of whom 171 were planned to undergo rotational atherectomy via radial and 256 via femoral access. During the three specified time intervals, the use of the transradial access for rotational atherectomy increased from 28 to 48% (Fig. 1).

Patients treated via radial access had a median age of 76 (IQR 68–81) and the majority (83.6%) were of male sex. Further baseline characteristics of patients treated via radial access are shown in Table 1. Acute coronary syndrome (ACS) was the indication for PCI in 12%, the majority of patients showed 3-vessel coronary artery disease (62%) and the LAD was the most common target vessel (49%).

**Table 1:** Baseline characteristics of all patients with planned rotational atherectomy

	Radial access (*n* = 171)	Femoral access (*n* = 256)	*p*-value
Male, %	83.6%	72.7%	0.080
Age (years), median (IQR)	76 (69–81)	75 (67–81)	0.560
Body mass index (kg/m^2^), mean ± SD	27.4 ± 5	27.8 ± 4	0.370
Hypertension, %	91.2%	95.7%	0.058
Hyperlipidemia, %	92.3%	92.5%	0.954
Prior or active smoking, %	28.1%	37.0%	0.055
Diabetes mellitus, %	29.2%	39.2%	**0.035**
History of CAD in family	17.0%	19.3%	0.542
Peripheral artery disease, %	15.2%	15.7%	0.879
Creatinine (mg/dl), mean ± SD	1.04 ± 0.51	1.38 ± 1.22	** < 0.001**
LV-EF (%), mean ± SD	51 ± 11	48 ± 14	** < 0.001**
Previous Stroke, %	8.2%	8.3%	0.976
Atrial fibrillation, %	22.8%	29.9%	0.105
Previous PCI, %	42.7%	52.9%	**0.038**
Previous CABG, %	9.9%	31.3%	** < 0.001**
Indications:			
Chronic coronary syndrome	87.7%	87.5%	0.946
NSTEMI	7.0%	7.1%	0.996
STEMI	0.6%	3.5%	0.05
Unstable angina	4.7%	2.0%	0.108
Extent of CAD			0.547
1-Vessel disease	11.1%	7.8%	
2-Vessel disease	26.9%	25.8%	
3-Vessel disease	62.0%	66.4%	
Target lesion			
LM	17.0%	21.1%	0.3
LAD	48.5%	34.4%	**0.003**
RCX	18.7%	23.8%	0.22
RCA	25.7%	34.4%	0.063
Bypass	0.6%	0.8%	0.816
RCX or RCA	44.4%	58.2%	**0.013**
Sheath size			** < 0.001**
6F	40.9%	16.4%	
7F	59.1%	76.6%	
8F	0.0%	7.0%	
Largest guiding size			** < 0.001**
6F	46.8%	25.4%	
7F	53.2%	71.1%	
8F	0.0%	3.5%	
Procedural success	97.1%	96.9%	0.83

*LV-EF* left ventricular ejection fraction, *PCI* percutaneous coronary intervention, *CABG* coronary artery bypass graft, *CAD* coronary artery disease, *LM* left main, *LAD* left anterior descending, *LCX* left circumflex, *RCA* right coronary artery

Compared to patients treated via femoral access, patients treated via radial access showed lower serum creatinine (1.04 ± 0.51 vs. 1.38 ± 1.22, *p* < 0.001) and a higher left ventricular ejection fraction (51% ± 11 vs 48% ± 14, *p* < 0.001), whereas patients treated via femoral access more often had a history of diabetes (39% vs. 29%, *p* = 0.035), prior PCI (53% vs. 43%, p = 0.038) or prior coronary artery bypass graft surgery (31% vs. 10%, *p* < 0.001).

### Procedural success

Overall procedural access of rotational atherectomy via the radial access was 97% (166/171). In one patient the wire and in 3 patients the burr could not be advanced through the stenosis. Of the latter 3 patients, two patients were treated using 6F guiding catheters, utilizing a 1.25 mm burr and a 1.5 mm burr in one case each. The third patient was treated via a 7F guiding catheter with a 1.5 mm burr. Only one patient showed a final TIMI flow < 3.

Success rate via the femoral approach was not significantly different (248/256; 97%; *p* = 0.99): 1 patient with ACS died by cardiogenic shock, after placement of the wire prior to performance of the rotational atherectomy, while in 4 patients the wire and in 2 patients the burr (1.75 mm via 7F catheter and 1.25 mm via 6F catheter) could not be advanced through the stenosis. 1 patient showed a final TIMI flow < 3.

### Procedural aspects

Overall, 11 patients were excluded from the analysis of procedural parameters since rotational atherectomy was ultimately not performed. Procedural data of all 416 patients, who underwent rotational atherectomy are shown in Table 2.

**Table 2:** Procedural characteristics of all patients, who underwent rotational atherectomy

	Radial access (*n* = 167)	Femoral access (*n* = 249)	*p*-value
ROTA/Diagnostic in same procedure	46.1%	27.7%	** < 0.001**
ROTA w/o previous dilatation	70.7%	63.9%	0.149
Sheath size			** < 0.001**
6F	39.5%	16.1%	
7F	60.5%	76.7%	
8F	0.0%	7.2%	
Pacemaker total	19.8%	36.1%	** < 0.001**
Permanent pacemaker	6.0%	8.8%	0.285
Temporary pacemaker	13.8%	27.3%	**0.001**
Extent of CAD			0.527
1-Vessel disease	10.8%	8.0%	
2-Vessel disease	27.5%	25.7%	
3-Vessel disease	61.7%	66.3%	
Treated Lesion			
LM	17.5%	21.7%	0.280
LAD	48.5%	34.1%	**0.003**
LCX	18.6%	23.7%	0.213
RCA	26.3%	34.5%	0.077
Bypass	0.6%	0.8%	0.809
LCX or RCA	44.9%	58.2%	**0.008**
ROTA > 1 vessel	10.8%	13.7%	0.619
Largest guiding size			** < 0.001**
6F	45.5%	24.1%	
7F	54.5%	72.3%	
8F	0.0%	3.6%	
Number of Burrs used			0.178
1	98.8%	97.6%	
2	0.6%	2.4%	
3	0.6%	0.0%	
Largest Burr (mm)			0.514
1.25	40.7%	43.8%	
1.5	45.5%	44.6%	
1.75	13.8%	10.8%	
2.00	0.0%	0.8%	
Largest Burr (mean ± SD)	1.43 ± 0.17	1.42 ± 0.174	0.527
Total stent length (mm)	59 ± 34	62 ± 34	0.425
DES Implantation	100.0%	98.8%	0.81
Application of DEB	7.2%	4.8%	0.256
Contrast agent (mean ± SD)	217 ± 91	217 ± 98	0.958
Fluoroscopy (hours; mean; SD)	0:24:51 ± 0:13:58	0:30:43 ± 0:19:18	** < 0.001**
DAP (µgm2)	9411 ± 6279	10,327 ± 7678	0.206
Duration (hours; mean; SD)	1:27:34 ± 0:34:53	1:36:50 ± 0:44:34	**0.018**
Additional IVL Treatment	0.0%	0.8%	0.246
Mechanical support (e.g., Impella)	0.0%	3.2%	**0.02**
TIMI flow III	99.4%	99.6%	0.95
Intrahospital death	0.6%	1.2%	0.535

*ROTA* rotational atherectomy, *CAD* coronary artery disease, *LM* left main, *LAD* left anterior descending, *LCX* left circumflex, *RCA* right coronary artery, *DES* drug-eluting Stent, *DEB* drug-eluting balloon, *DAP* dose-area product, *IVL* intravascular lithoplasty

**Fig. 2:**
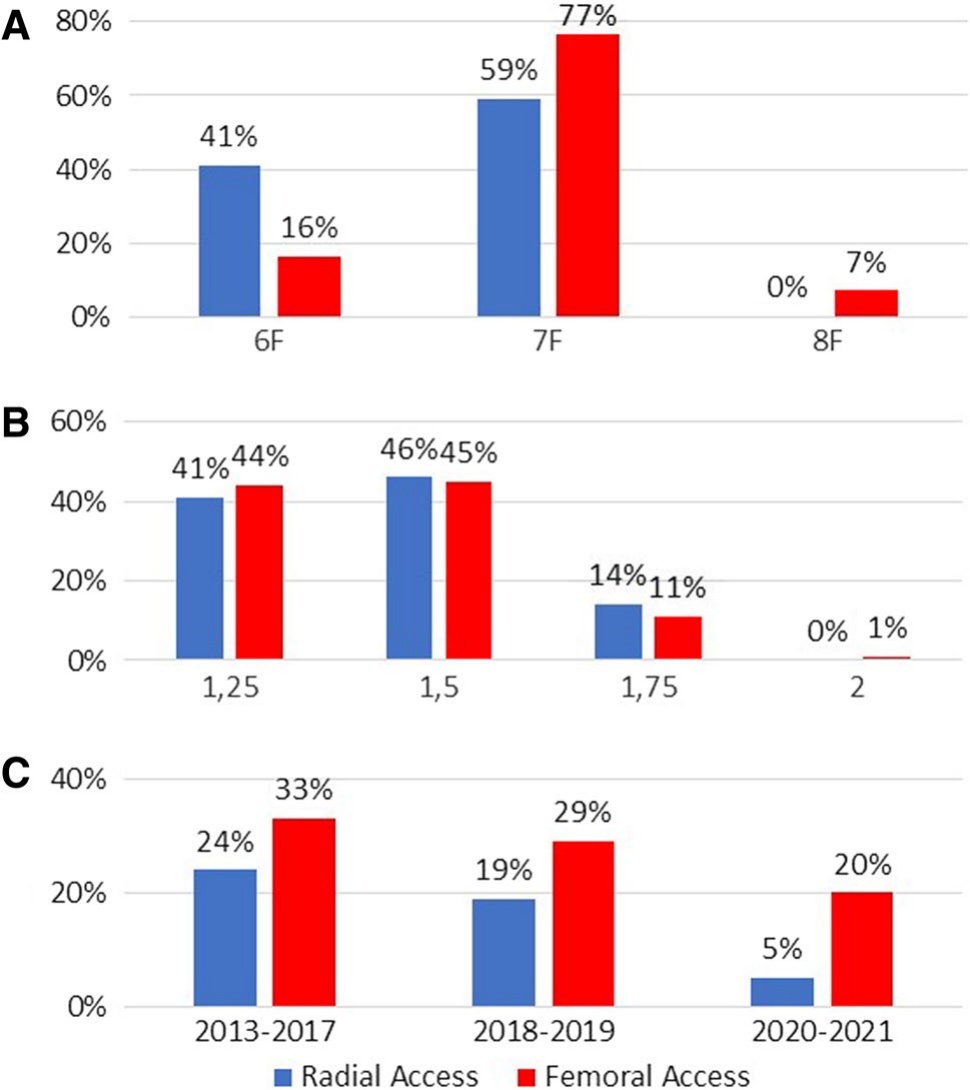
**A** Maximal sheath size in dependence of the access used in patients planned to undergo rotational atherectomy, **B** maximal burr sizes (mm) in dependence of the access used in patients, who underwent rotational atherectomy, **C** use of temporary pacemaker within the years 2013–2017, 2018–2019, 2020–2021 in patients undergoing rotational atherectomy via the radial or femoral access

In the majority of all patients treated via radial access (54%), rotational atherectomy was performed as a scheduled procedure. The LAD was the most frequently treated vessel (49%), and in 11% of patients, more than one vessel was treated. A 6F sheath was used in 41% of patients treated via the radial access, while a 7F sheath was used in 59% (Fig. 2A). Rotational atherectomy was performed via 6F guiding catheters in 47% and in 53% via 7F guiding catheters, with use of the 1.25 mm burr size in 41%, the 1.5 mm burr size in 46% and the 1.75 mm burr size in 14% (Fig. 2B). The use of temporary pacemakers in patients treated via the radial access declined from 24% in the years 2013–2017 to 5% in the years 2020–2021, with an overall use of a temporary pacemaker in 14% of the patients. Periprocedural complications occurred in 14.5% of patients and were mainly driven by hemodynamic instability which could be successfully treated through medication. There were no significant differences between the radial and femoral groups. (Table 3).

**Table 3:** Periprocedural complications of patients who underwent rotational atherectomy

	Radial access (*n* = 167) (%)	Femoral access (*n* = 249) (%)	*p*-value
Overall	14.5	17.7	0.386
Coronary perforation	1.2	2.0	0.537
Slow flow/No flow Phenomenon	0.6	0.8	0.816
Periprocedural ischemic ECG changes	3.6	3.6	0.994
Hemodynamic instability	7.8	10.4	0.379
Bradycardia	1.8	4.0	0.208
Burr/Wire complications	0.0	2.0	0.077
Periprocedural death	0.0	0.4	0.415

Patients in the femoral group were less often treated ad hoc than patients in the radial group (28% vs radial 46%, *p* < 0.001). The LAD and RCA were equally often the targeted vessel (34% each), without significant difference compared to patients treated via radial access (*p* = 0.077). Analyzing LCX and RCA together, these vessels were more often the target vessels in the femoral group (58% vs. 44%; *p* = 0.013).

Whereas sheath and guiding sizes were smaller in the radial group (*p* < 0.001; Table 2), burr sizes did not differ between the groups (*p* = 0.51). Insertion of a temporary pacemaker was more frequent in the femoral group (27% vs. 14%, *p* < 0.001), but also declined over the years (Fig. 2C). Mechanical circulatory support was only used in the femoral group (3.2% vs. 0%, *p* = 0.02). The fluoroscopy time (30:43 ± 19:18 Min vs. 24:51 ± 13:58 min; *p* < 0.001) as well as the duration of the whole procedure (1:37 ± 0:45 h vs 1:27 ± 0:35 h vs.; *p* = 0.018) took longer in patients of the femoral group.

### Access site-related complications

After the exclusion of 8 patients in which mechanical circulatory support was used, 419 patients, who were scheduled for rotational atherectomy, were analyzed for access-related complications. Overall, access site-related complications occurred in 9% of these patients (radial 4% vs femoral 13%, *p* = 0.003; Table 5). 3 patients showed either aneurysms or ischemia simultaneously with bleeding complications.

Whereas patients with access-related complications showed higher left ventricular function (53 ± 11% vs. 49 ± 13%, *p* = 0.037) in the univariate analysis, only femoral access (OR 3.3; 95% CI 1.4–8.0, *p* = 0.007) and female sex (OR 3.4; 95% CI 1.7–6.8) showed independent predictive value for access site-related complications (Table 4).

**Table 4:** Characteristics of patients with access-related complications and patients without access-related complications after exclusion of patients treated with mechanical support

	Access-related complications (*n* = 38)	No access-related complications (*n* = 381)	*p*-value
Gender (male)	50.0%	79.4%	< 0001
Age (median; IQR)	76 (68–81)	76 (68–81)	0.827
BMI (mean; SD)	27.3 ± 4.1	27.8 ± 4.6	0.495
Diabetes	21.1%	36.4%	0.058
Art. Hypertension	92.1%	93.9%	0.657
Atrial fibrillation	34.2%	26.7%	0.319
PAD	7.9%	16.6%	0.160
Patient on ASS	68.4%	76.8%	0.250
Patient on DAPT	26.3%	30.1%	0.122
Oral Anticoagulation	23.7%	24.5%	0.907
LV function	53 ± 11%	49 ± 13%	0.037
Creatinine (mg/dl)	1.18 ± 1.09	1.25 ± 1.03	0.730
Procedural Characteristics			
ACS	13.2%	12.4%	0.893
Femoral access	81.6%	56.9%	0.003
Sheath size			0.672
6F	21.1%	26.9%	
7F	76.3%	69.4%	
8F	2.6%	3.7%	
Temporary Pacemaker	21.1%	21.9%	0.904
Intrahospital Death	2.6%	1.1%	0.395

*BMI* body mass index, *PAD* peripheral arterial disease, *ASS* acetylsalicylic acid, *DAPT* dual antiplatelet therapy, *LV* left ventricular, *ACS* acute coronary syndrome

Bleeding occurred overall in 8% of patients and was more often present in patients treated via the femoral access (11% vs. 4%, *p* = 0.006). Only in the femoral group, BARC 3a (*n* = 10) and BARC 3b (*n* = 3) bleeding events as well as the need for transfusion (*n* = 5) were observed. The drop of hemoglobin in patients with bleeding complications prior and after the procedure was higher in patients in the femoral group, albeit not statistically significantly different from the radial group (femoral 2.7 g/dl (+ − 1.9 g/dl) vs. radial 0.8 g/dl (+ − 1.1 g/dl); p = 0.10). Aneurysms, AV fistulas and ischemia were very rare in the whole population (Table 5).

**Table 5:** Access-related complications in patients planned to undergo rotational atherectomy either via radial or femoral access

	Radial access (*N* = 171)	Femoral access (*n* = 256)	*p*-value
Access-related complications overall	4.1%	12.5%	**0.003**
Bleeding	3.5%	10.9%	**0.005**
BARC Classification			**0.013**
BARC 2	100.0%	50.0%	
BARC 3a	0.0%	35.7%	
BARC 3b	0.0%	14.3%	
Transfusion	0.0%	17.9%	0.26
Delta Hb (mean ± SD)	0.78 (± 0.81)	2.81 (± 1.59)	**0.004**
Aneurysm	0.6%	1.6%	0.64
AV fistula	0.0%	0.8%	0.66
Ischemia	0.0%	0.4%	0.84

## Discussion

Our analysis including 427 patients undergoing rotational atherectomy illustrates:

1. Radial access for rotational atherectomy became more common throughout the years and has been used in nearly half of the all procedures during the last third of the observation period. Having no difference in success rate as compared to femoral access.
2. Temporary pacemakers as well as the use of mechanical support were more often present in the femoral group. The majority of all patients treated with rotational atherectomy was treated with either a 6F or 7F sheath and guiding catheters. The use of a burr ≥ 2.0 mm was rarely necessary.
3. Patients treated with rotational atherectomy via radial access showed a significant lower rate of access site related complications, as well as severe bleeding events. In addition, femoral access was a predictor for access-related complications.

### Current use of access site in patients undergoing rotational atherectomy

With the aging of society and an increased number of patients undergoing invasive coronary procedures, the incidence of severely calcified coronary stenoses is on the rise and will continue to do so. Together with the expansion of PCI indications over the last years, interventional treatment of severely calcified stenoses has become far more common [1, 3]. While, currently only 1–2% of PCIs are performed with rotational atherectomy [1, 2, 10], the number of atherectomy procedures is expected to increase significantly in the near future.

While clinical guidelines clearly recommend the radial access route for PCI in general due to lower mortality and MACE rates [3, 11], the recommendation for access sites in case of rotational atherectomy, both in European as well as in North American expert consensus documents, is far more conservative [1, 2]. Nevertheless, the percentage of radial access in patients undergoing rotational atherectomy, confirmed by our recent as well as previous data [12], has grown over the past years. This is possibly due to increasing expertise in transradial PCI in the interventional community, in particular after transradial PCI became the recommended standard approach [3]. Still, radial access is nowadays used only in 50–60% of all cases undergoing rotational atherectomy cases—both in our cohort and in previously published data. [4, 7, 12].

### Procedural aspects of rotational atherectomy via the radial access

In clinical practice, procedural factors have an influence on the decision of the access route for rotational atherectomy. The most important considerations might be the concomitant use of a temporary pacemaker or mechanical circulatory support, followed by the possible need to use a larger burr sizes which in turn require larger guiding catheters.

#### Target vessel and the use of a temporary pacemaker

The use of a temporary pacemaker remains frequently recommended for patients undergoing rotational atherectomy of the RCA or of a dominant LCX [1]. This is based on the assumption, that these vessels provide the major circulation for the cardiac conduction system and rotational atherectomy of these vessels might cause high-degree atrio-ventricular blocks [13].

Concerning the temporary pacemaker insertion via the femoral vein, it seems convenient to also perform the procedure itself via the femoral access. The treatment of RCA and LCX and especially the use of a temporary pacemakers is therefore more frequent in patients treated via femoral access both in our as well as in previously published data. [4, 6, 7] Use of a temporary pacemaker has been reported as high as 65% for patients treated via the femoral access [4, 6, 7]. However, this approach should be questioned: On one hand, the additional insertion of a pacemaker via the femoral vein does not necessarily constitute an exclusion criterion for performing PCI via the radial access route. In our cohort, 14% of patients treated via the radial access also received a temporary pacemaker. On the other hand, pacemaker placements may not be routinely necessary even when treating the RCA or a dominant LCX, given the temporary nature of the induced AV block [4]. In fact, the use of a temporary pacemaker in rotational atherectomy currently is subject to pronounced regional differences. While in the large national UK registry study a temporary pacemaker was used in less than 1% of all patients, our cohort was defined by a percentage of 22%, whilst other registries had a percentage as high as 36%. [4, 6, 7]. Recent experience has shown that conduction disturbances during rotational atherectomy are not as common as initially thought [2]. Additionally, they might be prevented by medication alone, e.g., with the administration of atropine or aminophylline [2, 14]. Our data confirms a steep decrease in the use of a temporary pacemakers over the past years. However, randomized data on this question are missing and expert recommendations have not been revised [1], which results in large variability of concomitant pacemaker use between individual centers.

#### Concomitant mechanical circulatory support

The insertion of mechanical support in most cases requires a large femoral access. Therefore, unilateral or bilateral femoral access is often used for patients undergoing protected PCI. This is evident in our results, where all patients in which rotational atherectomy was performed with concomitant mechanical support were treated via the femoral access. Again, data from the UK registry show that this is not necessarily required. Here, too, mechanical support was used more frequently in patients with femoral access, but also in 1.6% of patients who underwent rotational atherectomy via the radial access [4]. According to the data that show clear advantages of radial access for PCI [15–17], but also data implying an advantage of combining radial and femoral access rather than using bilateral femoral access [18], rotational atherectomy with concomitant mechanical support should either be performed via radial access or better via the sheath of the mechanical support to avoid any additional access.

#### Guiding and burr sizes

As demonstrated by the STRATAS and CARAT studies, smaller burrs with a burr to artery ratio < 0.7 permit angiographic and procedural success equivalent to that of larger burrs [19, 20]. Thus, plaque modification is easily achieved with a 1.25 mm or a 1.5 mm burr in nearly all cases [6, 19, 20]. Additionally, smaller burrs enable the use of smaller sheaths and guiding catheters and show fewer complications [19, 20].

Our data confirm that most rotational atherectomy cases are performed with either 1.25 mm or 1.5 mm burrs—independent of the access site. Only about 12%—interestingly numerically more patients in the radial group—were treated with a 1.75 mm burr and only 0.5% with a 2.0 mm burr. Thus, in our cohort, 87% of patients undergoing rotational atherectomy could have been treated with a 6F and 99.5% with a 7F guiding catheter. Nevertheless, the preferred sheath size for rotational atherectomy in our cohort was still 7F—independent of the access site.

Here, two factors are predominantly responsible: First, a better initial situation in the event of serious complications such as coronary perforations. Second, the higher comfort of using a bigger guiding catheter for advancing the burr to the vessel.. To the first factor can be said, that serious complications in which a bigger guiding size would be of use, were very rare and could all be handled via a 6F guiding catheter. The second factor should be addressed in more detail: A 7F sheath as well as 7F guiding catheters are commonly and safely used in radial access—if necessary. Especially in a complex lesion, a 7F radial approach showed lower rates of vascular complications than 7F femoral approach, without having any difference in procedural success, duration, radiation or contrast volume [21]. Still, one should keep in mind that bigger sheath sizes are associated with higher rates of radial artery occlusion [22]. Thus, in the only small percentage of patients, in which a 7F guiding catheter is really necessary, thin-walled radial introducer sheaths (= slender sheaths) or sheath-less options seem a very good alternative to use 7F guiding catheters without increasing the diameter of the inserted sheath.

### Access site-related complications

Radial access is now the preferred and recommended access for PCI in general [3]. This is based on a reduced mortality and lower bleeding complications shown for patients undergoing PCI, especially in the scenario of an acute coronary syndrome [15–17]. Modifiable risk factors for bleeding complications are the size of the vascular sheath and the doses of heparin or the use of GP IIb/IIIa inhibitors [23]. Non-modifiable factors known to elevate the bleeding risk in patients undergoing PCI are old age, a female sex, hypertension, anemia, renal insufficiency as well as signs for acute coronary syndrome [23]. As patients with heavy calcified stenoses are primarily of older age and more often showing renal insufficiency [24] and are treated with bigger sheath sizes [7, 8], these patients seem at a particularly high risk for bleeding. With an overall rate of 9% for access-related complications, our data confirm data from previous studies and show the need to reduce this number dramatically. The total lack of stronger bleeding complications as BARC 3a or 3B as well as no single need for transfusion in the radial group of our cohort—even if only in a retrospective, non-randomized cohort—underlines the advantage of radial access in this population with an already fundamentally increased risk of bleeding. The predictive value of femoral access for access-associated complications in our cohort is an interesting phenomenon, but due to the limitation that no randomized cohort is described, it has to be considered as a mere hypothesis.

### Limitations

The data presented here were collected purely retrospectively. Since the decision on the access route was independently made by the operator, factors such as the use of a pacemaker or a mechanical support system certainly played a role and make a comparison of the radial with the femoral access route of limited predictive use. Nevertheless, the data clearly highlight the safety of the radial approach and that, especially when viewed retrospectively, almost all patients could have been treated via radial access. It also needs to be remarked, that we cannot provide data on periprocedural myocardial infarction, as we do not routinely determine troponin after each PCI. In addition, the assay was changed during the observation period, which further confounds any further interpretation.

## Conclusions

In recent years, the use of radial access for rotational atherectomy has been increasing. Burr sizes used in clinical daily routine allow the use of a 6F sheath and guiding catheters in the majority of cases. If the femoral approach is used, e.g., to facilitate insertion of a temporary pacer, the patient's risk of access-related complications seems to increase substantially. Severe bleedings—BARC 3a, BARC 3b—were only observed in patients treated via femoral access.

Hence, the advantages and low access complications rates ought to be kept in mind, even when requiring a 7F guiding or a temporary pacemaker radial access should be considered for rotational atherectomy. Data from randomized trials or big propensity-matched cohorts could help to make clear recommendations from the merely hypothesis-generating data available from our study.

## Abbreviations

LAD: Left anterior descending artery
LCX: Left circumflex artery
RCA: Right coronary artery
PCI: Percutaneous coronary intervention
ECG: Electrocardiogram
BARC: Bleeding Academic Research Consortium
BMI: Body mass index
ACS: Acute coronary syndrome
LV: Left ventricle
DAPTV: Dual antiplatelet therapy
PAD: Peripheral artery disease
